# What drives polyploidization in plants?

**DOI:** 10.1111/nph.15929

**Published:** 2019-06-12

**Authors:** Tod Stuessy, Hanna Weiss‐Schneeweiss

**Affiliations:** ^1^ Department of Botany and Biodiversity Research University of Vienna Rennweg 14 A‐1030 Vienna Austria; ^2^ Herbarium and Department of Evolution, Ecology, and Organismal Biology The Ohio State University 1315 Kinnear Avenue Columbus OH 43212 USA

**Keywords:** allopolyploidy, chromosome doubling, evolution, genetic distance, hybridization, speciation

## Abstract

This article is a Commentary on Wagner *et al*. **223**: 2039–2053.

Organisms with more than two full sets of chromosomes occur throughout many different taxonomic groups, but they are especially prevalent among ferns and flowering plants. The ferns are known to have very high ploidy levels, including the highest known 2*n* = 1440 in *Ophioglossum reticulatum* (Khandelwal, 1990). The flowering plants reflect a broad range of chromosome numbers (Weiss‐Schneeweiss & Schneeweiss, 2013) from 2*n* = 4 to 2*n* = 640. More importantly, surveys of levels of polyploidy among flowering plants have estimated that at least 35% of present species within genera are recent polyploids (Wood *et al*., 2009). Because of the high level of occurrence of polyploidy in vascular plants, it must be the case that polyploidy has been associated with speciation and is, therefore, of substantial evolutionary significance. Despite considerable investigations on the mechanisms and consequences of polyploidy, both taxonomic and evolutionary, much less is known about the causes of polyploidy (Soltis *et al*., 2010). Why the phenomenon has been so common in certain groups and not in others is poorly understood. The formation of polyploids does correlate with some environmental factors, such as latitude and elevation (i.e. lower temperatures), a correlation known for decades (e.g. Ehrendorfer, 1980), and this has recently been confirmed with more precise data (e.g. *Ranunculus*, Ranunculaceae; Schinkel *et al*., 2016). In this issue of *New Phytologist*, Wagner *et al*. (pp. 2039–2053), however, looks at the reasons for propensity for polyploidization from an interesting genetic perspective within a phylogenetic context.

*‘The results of the current study provide arguments supporting … an important role of genetic distances in promoting allopolyploidization.’*

In any discussion of polyploidy, it is necessary to clarify the different types of mechanisms that result in chromosome set multiplication. The two general mechanisms are autopolyploidy and allopolyploidy (Fig. 1). The former is the simplest way for a chromosomal set to be multiplied, and this occurs within the same individual plant (through somatic doubling by failure of chromosomes to separate during mitosis) or, more frequently, by crosses within the same population (or individual) involving unreduced gametes, yielding polyploid offspring. Autopolyploids are known to be common within plant species, and numerous examples of polyploid cytological races exist (e.g. in *Melampodium*, Asteraceae; Stuessy *et al*., 2004). Although it was initially thought that autopolyploids would have little chance of leading to speciation because they are so genetically similar to the parents and with meiotic problems leading to reduced fertility, it is now acknowledged that autopolyploidization may be more common than realized previously (Parisod *et al*., 2010). The more frequent mode of polyploid formation, allopolyploidy, is via hybridization between two parents that have different adaptive norms, i.e. often different species, accompanied by chromosome doubling. This mechanism produces polyploid offspring that have genetic compositions different from either parent, and often different from the sum of the parents, which allows them to establish successfully and colonize new ecological niches. Numerous studies have revealed that allopolyploid species may have different ecological tolerances from their diploid progenitors (Ramsey, 2011). Furthermore, the multiplication of chromosome sets provides each chromosome with a homolog for often successful instantaneous bivalent pairing in meiosis, which has a better chance of resulting in fertile offspring.

**Figure 1:**
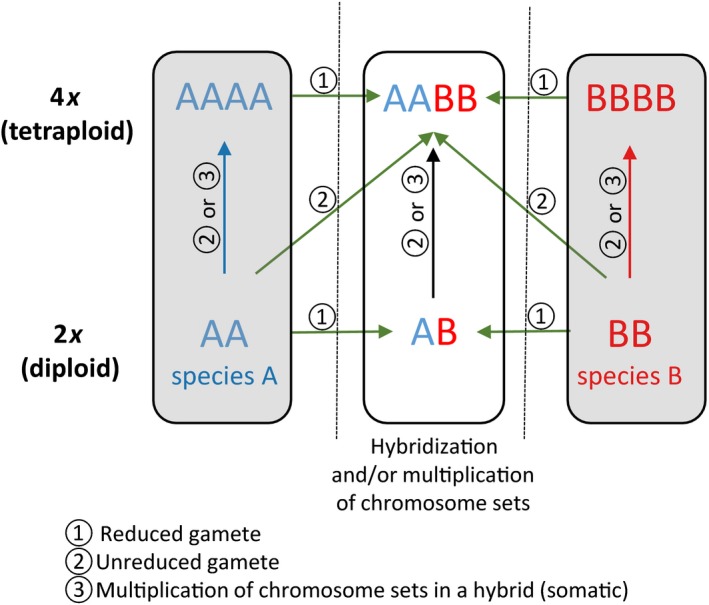
Simplified scheme of the potential pathways leading to the formation of autotetraploids and allotetraploids. Basic set of chromosomes of parental species A; basic haploid set of chromosomes of parental species B. Green arrows indicate hybridization; blue and red arrows, multiplication of chromosome sets within one species/taxon; black arrows, multiplication of chromosome sets of the homoploid diploid hybrid. Gray boxes show autopolyploidization; white box, allopolyploidization. For simplicity, polyploidization via triploid bridge is not shown.

To determine factors that influence propensity toward polyploidization, Wagner *et al*. have taken the approach of examining two closely related genera, one consisting of all diploid species and the other containing both diploid and polyploid species. The idea was to determine the degree of genetic diversity among diploid species of both genera as well as their proclivity for interspecific hybridization. Wagner *et al*. selected diploid species of two closely related genera of the sunflower family (tribe Anthemideae, subtribe Leucantheminae): *Leucanthemum* from Europe and *Rhodanthemum* from North Africa. The species of *Rhodanthemum* are all diploid, whereas those in *Leucanthemum* are both diploids and polyploids (more than 25 species; 4*x* to 22*x*). The authors are testing the hypothesis that hybridization is more common between species that are sufficiently genetically similar so that they can cross, but also genetically distinct enough to prevent homoeologous chromosome pairing and multivalent formation during meiosis in offspring, which can lead to sterility or reduced fertility in the gametes (Buggs *et al*., 2011).

To assess genomic divergence and traces of hybridization, Wagner *et al*. used several molecular markers and analytical approaches. For evaluation of phylogenetic Bray–Curtis genetic distance (Göker & Grimm, 2008) between diploid species of both genera, nucleotide sequences from eight nuclear single‐copy markers plus internal transcribed spacer (ITS) and also five cpDNA intergenic spacer regions were examined. JML and gene‐tree *gsi* (genealogical sorting index) were employed for detection of hybridization. To provide a time frame for the evolutionary events, a molecular clock was calibrated using *Beast (Heled & Drummond, 2010) for the phylogenies within both genera. The evaluations of molecular clock assessments relied on previous phylogenetic estimates of molecular divergence within Anthemideae along with fossil pollen data from *Artemisia*.

The data revealed that the diploid species of *Leucanthemum* are clearly more divergent among each other than those in *Rhodanthemum*. The data also showed that the diploid species of *Leucantheumum* carried more genomic signatures of past interspecific hybridization events than did those of *Rhodanthemum*. The importance of genetic divergence as a stimulus for hybridization had been proposed by Darlington (1937), and it has since been regarded as controversial (Paun *et al*., 2009; Buggs *et al*., 2011). The results of the current study provide arguments supporting the early hypothesis, suggesting an important role of genetic distances in promoting allopolyploidization.

The remaining question was why did the species of *Leucanthemum* hybridize so frequently? A map of present distribution of the genus reveals localization in many of the previous refugial areas in Europe during the Pleistocene. As Wagner *et al*. suggest, the changing climate during the Pleistocene may have led to secondary contacts between species, which in view of the genetic distances previously discussed, might have led to allopolyploidization. The species of *Rhodanthemum* southward in the Atlas mountains of Morocco would have been much less affected by Pleistocene climatic changes, and this, plus the lower genetic divergence among species, may explain the absence of polyploids in this lineage.

The important conclusion by Wagner *et al*. is that we now have a specific hypothesis for the formation of allopolyploids within particular groups of flowering plants, which can be tested in other genera. The challenge will be to select systems in which closely related sister genera differ in amounts of diploid and polyploid species, so that genetic divergence and tendencies for hybridization can be examined in each and compared. The tendency for allopolyploidization is clearly influenced by various environmental and genetic factors that are often acting in synergy and hence difficult to disentangle. The article by Wagner *et al*. provides clear evidence that genetic divergence among diploid species is indeed one of these significant stimuli and should always be considered when analyzing causes of allopolyploidization in other groups of species.

## References

[nph15929-bib-0001] Buggs RJA , Soltis PS , Soltis DE . 2011 Biosystematic relationships and the formation of polyploids. Taxon 60: 324–332.

[nph15929-bib-0002] Darlington CD . 1937 Recent advances in cytology, *2*^*nd*^*edn* Philadelphia, PA, USA: Blakiston.

[nph15929-bib-0003] Ehrendorfer F . 1980 Polyploidy and distribution In: Lewis WH, ed. Polyploidy: biological relevance. New York, NY, USA: Plenum Press, 45–60.

[nph15929-bib-0004] Göker M , Grimm GW . 2008 General functions to transform associate data to host data, and their use in phylogenetic inference from sequences with intra‐individual variability. BMC Evolutionary Biology 8: 86. 18366660 10.1186/1471-2148-8-86 PMC2291458

[nph15929-bib-0005] Grant V . 1981 Plant speciation, *2*^*nd*^*edn* New York, NY, USA: Columbia University Press.

[nph15929-bib-0006] Heled J , Drummond AJ . 2010 Bayesian inference of species trees from multilocus data. Molecular Biology and Evolution 27: 570–580. 19906793 10.1093/molbev/msp274 PMC2822290

[nph15929-bib-0007] Khandelwal S . 1990 Chromosome evolution in the genus *Ophioglossum* L. Botanical Journal of the Linnean Society 102: 205–217.

[nph15929-bib-0008] Parisod D , Holderegger R , Brochman C . 2010 Evolutionary consequences of autopolyploidy. New Phytologist 186: 5–17. 20070540 10.1111/j.1469-8137.2009.03142.x

[nph15929-bib-0009] Paun O , Fay M , Soltis DE , Chase MW . 2009 Hybrid speciation in angiosperms: parental divergence drives ploidy. New Phytologist 182: 507–518. 19220761 10.1111/j.1469-8137.2009.02767.x PMC2988484

[nph15929-bib-0010] Ramsey J . 2011 Polyploidy and ecological adaptation in wild yarrow. Proceedings of the National Academy of Sciences, USA 108: 7096–7101. 10.1073/pnas.1016631108 PMC3084070 21402904

[nph15929-bib-0011] Schinkel CF , Kirchheimer B , Dellinger AS , Klatt S , Winkler M , Dullinger S , Höerandl E . 2016 Correlations of polyploidy and apomixis with elevation and associated environmental gradients in an alpine plant. AoB Plants 8: plw064. 27594702 10.1093/aobpla/plw064 PMC5091893

[nph15929-bib-0012] Soltis DE , Buggs RJA , Doyle JJ , Soltis PS . 2010 What we still don't know about polyploidy. Taxon 59: 1387–1403.

[nph15929-bib-0013] Stuessy TF , Weiss‐Schneeweiss H , Keil DJ . 2004 Diploid and polyploid cytotype distribution in *Melampodium cinereum* and *M. leucanthum* (Asteraceae, Heliantheae). American Journal of Botany 91: 889–898. 21653445 10.3732/ajb.91.6.889

[nph15929-bib-0014] Wagner F , Ott T , Zimmer C , Reichhart V , Vogt R , Oberprieler C . 2019 ‘At the crossroads towards polyploidy’: genomic divergence and extent of homoploid hybridization are drivers for the formation of the ox‐eye daisy polyploid complex (*Leucanthemum*, Compositae‐Anthemideae). New Phytologist 223: 2039–2053. 30851196 10.1111/nph.15784

[nph15929-bib-0015] Weiss‐Schneeweiss H , Schneeweiss G . 2013 Karyotype diversity and evolutionary trends in angiosperms In: Leitch IJ, Greilhuber J, Doležel J, Wendel JF, eds. Plant genome diversity Volume 2: physical structure, behaviour and evolution of plant genomes. Wien, Germany: Springer‐Verlag, 209–230.

[nph15929-bib-0016] Wood TE , Takebayashi N , Barker MS , Mayrose I , Greenspoon PB , Rieseberg LH . 2009 The frequency of polyploid speciation in vascular plants. Proceedings of the National Academy of Sciences, USA 106: 13875–13879. 10.1073/pnas.0811575106 PMC2728988 19667210

